# Electrochemical sensors for the determination of 4-ethylguaiacol in wine

**DOI:** 10.1007/s00604-023-05729-8

**Published:** 2023-03-18

**Authors:** Paula Portugal-Gómez, A. Marta Navarro-Cuñado, M. Asunción Alonso-Lomillo, Olga Domínguez-Renedo

**Affiliations:** Faculty of Sciences, Analytical Chemistry Department, University of Burgos, Pza. Misael Bañuelos S/N, 09001 Burgos, Spain

**Keywords:** 4-Ethylguaicol, Screen-printed electrodes, Differential pulse voltammetry, Fullerene, Wine

## Abstract

The development of an electrochemical procedure for the determination of 4-ethylguaiacol and its application to wine analysis is described. Modified screen-printed carbon electrodes (SPCEs) with fullerene C_60_ (C_60_) have been shown to be efficient in this kind of analysis. The developed activated C_60_/SPCEs (AC_60_/SPCEs) were adequate for the determination of 4-ethylguaicol, showing a linear range from 200 to 1000 µg/L, a reproducibility of 7.6% and a capability of detection (CC_*β*_) value of 200 µg/L, under optimized conditions. The selectivity of the AC_60_/SPCE sensors was evaluated in the presence of possibly interfering compounds, and their practical applicability was demonstrated  in the analysis of different wine samples obtaining recoveries ranging from 96 to 106%.

**Graphical Abstract:**

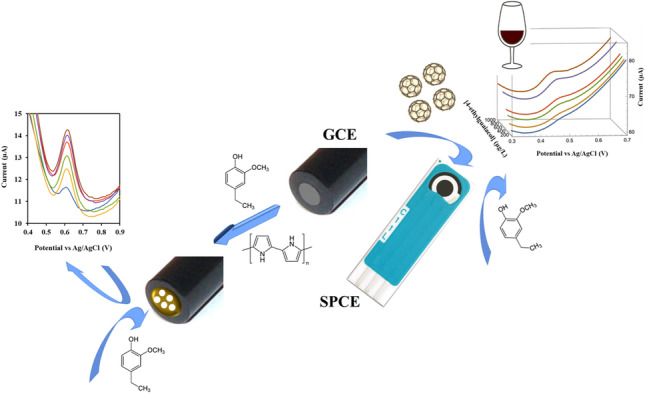

## Introduction

The occurrence of unpleasant odours in wine that negatively affect its quality may be related to the presence of certain molecules, including volatile phenols, such as 4-ethylguaiacol, associated to the advent of smoked aromas [1, 2]. Therefore, the development of analytical methods that allow the early detection of this compound is extremely important to avoid the rejection of a wine, which seriously affects the reputation and economy of the producing winery.

Most of the methods described for the quantification of 4-ethylguaicol in wine are based on the use of chromatographic techniques, being gas chromatography using mass spectrometric [3–10] and flame ionization [11–15] detection systems the most often selected technique in the analysis of this compound due to its volatility [3–16]. Wine is a complex matrix with many different constituents present at different concentration levels, considered low in the case of volatile phenols (between 1 and 2660 µg/L) [4]. Thus, the measurement of 4-ethylguaiacol in wine using gas chromatographic techniques frequently involves previous sample preparation steps based on different extraction procedures, including liquid–liquid extraction [3, 4], solid-phase extraction [10, 11, 14, 15] and stir bar sorptive extraction [8, 9]. Liquid chromatography has also been selected as an analytical technique in the determination of 4-ethylguaiacol in wine using diode array [17, 18] and fluorescence [17, 19] detectors, although the number of methods developed is considerably lower compared to gas chromatography. These chromatographic procedures also involve the use of preparative steps based on solvent and solid-phase extraction processes. The main advantages of the described chromatographic techniques are their high precision, selectivity and sensitivity. However, these techniques are associated with high-cost laboratory equipment and highly qualified personnel requirements, being difficult to adapt for in situ measurements, for example in a winery during the wine production process [16].

Electrochemical sensors may be an interesting alternative to chromatographic techniques in the determination of 4-ethylguaiacol since they can easily be oriented towards in situ analysis, being able to detect the cause of the contamination just at the moment it occurs with also high sensitivity and selectivity [20, 21]. Like this, 4-ethylguaicol has been analysed using different electrochemical techniques (Table 1) including screen-printed carbon electrodes (SPCEs) modified with TiO_2_ or SnO_2_ nanoparticles [22]. The joint determination of different phenols has also been approached using electrochemical sensors. Thus, the analysis of 4-ethylguaiacol in the presence of other phenols can be carried out using Nafion-modified boron-doped diamond electrodes in the presence of α-cyclodextrin [23] or by means of an array of different modified electrodes using artificial neural networks for quantitative analysis [20, 24, 25]. Despite the advantages of the described electrochemical methods, only one of these works involves the determination of 4-ethylguaicol in wine, but the detection limit reached (5.5 mg/L) above the normal content in wine samples [20]. Thus, the electrochemical analysis of this compound in complex samples, such as wine, requires the development of more sensitive and selective devices. In this way, highly selective sensors for the analysis of other volatile compounds in wine have been established by means of the modification of the working electrode surface with a molecularly imprinted polypyrrole polymer [26]. However, experiments carried out with this type of modification in this work, using 4-ethylguaicol as target molecule, did not lead to satisfactory results in its analysis in wine samples. On the other hand, the modification of the working electrode with nanomaterials has already proven to be suitable for the development of sensors for the analysis of similar compounds due to its capability to accept and donate electrons [27]. Among them, fullerene C_60_ (C_60_) has demonstrated to be an excellent material for electrode modification due to its high electroactive surface area and good conductivity. Moreover, C_60_-modified electrodes have shown a long stability and a wide potential window [28]. Therefore, disposable SPCEs have been modified with C_60_ for the development of sensitive and selective suitable sensors for the determination of 4-ethylguaiacol in wine. In addition, a selectivity improvement has been carried out in these analyses through a previous accumulation step of the analyte present in the gas phase set over the liquid sample [27].

**Table 1:** Electrochemical sensors for 4-ethylguaicol

Technique	Electrode	Limit/capability of detection (µg/L)	Reproducibility (%)	Sample	Recovery (%)	Reference
CV	Array of 6 GECEs modified with different materials	5500	6.5	Wine	–	[20]
	SPCE modified with SnO_2_ nanoparticles	12.5	–	–	–	[22]
	SPCE modified with TiO_2_ nanoparticles	19.2				
	CPE	16.9	–	–	–	[24]
	CPME-CNT	16.1				
	CPME-AB	14.3				
DPV	SPCE modified with SnO_2_ nanoparticles	9.4	2.48	Simulated chemical mixture of volatiles	91.6–108.8	[22]
	SPCE modified with TiO_2_ nanoparticles	5.3	4.85		91.0–101.8	
	Array of 5 GECEs modified with MIPs	2400	–	–	–	[25]
	AC_60_/SPCE	200	7.6	Wine		This work
SWV	Nafion-modified BDDE	15.2	–	Whiskey samples	–	[23]

*AC*_*60*_*/SPCE* activated fullerene-modified screen-printed electrode, *BDDE* boron-doped diamond electrode, *CPE* carbon paste electrode, *CPME-CNT* carbon paste–modified electrode with carbon nanotubes, *CPME-AB* carbon paste–modified electrode with activated biochar, *CV* cyclic voltammetry, *DPV* differential pulse voltammetry, *GECEs* graphite epoxy composite electrodes, *MIPs* molecularly imprinted polymers, *SWV* square wave voltammetry

## Experimental

### Reagents

Reagents used were analytical reagent grade chemicals, and all solutions were prepared using Milli-Q water (18.2 MΩ/cm; Millipore, Bedford, MA, USA). 4-Ethylguaicol was purchased from Alfa Aesar (98%; Haverhill, MA, USA); 0.1 M phosphate (KH_2_PO_4_) buffer solutions (Fluka, Munich, Germany) containing 0.1 M potassium chloride (Merck, Darmstadt, Germany) were used as supporting electrolyte for the electrochemical measurements; 1 M phosphoric acid (85%; Panreac, Barcelona, Spain) was used to adjust the pH of the buffer solutions to the adequate value; C_60_ solutions (99.9%; Acros Organics, Geel, Belgium), used in the C_60_/SPCE generation, were prepared using dichloromethane, purchased from Panreac (Barcelona, Spain), as solvent; 1.0 M KOH solutions (Carlo Erba, Val de Reuil, France) were used in the reduction of these modified electrodes; and 4-ethylphenol (97%; Alfa Aesar, Haverhill, MA, USA), 4-vinylphenol (10 wt% solution in propylene glycol; SAFC, St. Louis, MO, USA) and *p*-coumaric acid (> 98%; Sigma-Aldrich, Steinheim, Germany) were analysed as possible interferences.

### *Preparation of AC*_*60*_*/GCEs*

**Fig. 1:**
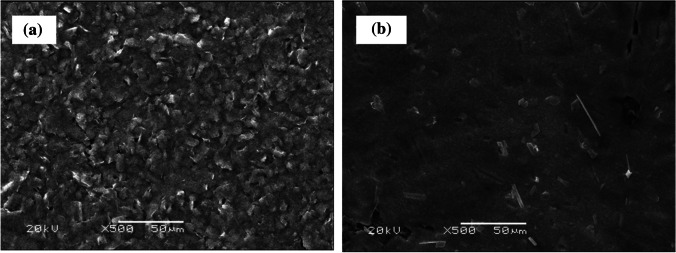
SEM images obtained for a SPCE (**a**) and an AC_60_/SPCE (**b**) with × 500 enlargement

The modification of the SPCE surface (DRP-C11L; Metrohm DropSens, Oviedo, Spain) with a well-coated layer of C_60_ (i.e. C_60_/SPCE) was performed according to a previously described procedure [27]. Briefly, 40 µL of 0.1 mg/mL solution of C_60_, prepared in dichloromethane, was deposited on the SPCE surface and allowed to dry at room temperature. This C_60_ film formed on the electrode surface was next partially reduced in 1.0 M KOH by cyclic voltammetry in the potential range from 0.0 to − 1.5 V vs. Ag/AgCl, at a scan rate of 10 mV/s, becoming conductive due to the formation of K_3_C_60_ salt [29]. Thus, an activated C_60_/SPCE (AC_60_/SPCE) was obtained in this way. The formation of the AC_60_ layer on the SPCE can be seen in the scanning electron microscope (SEM) images shown in Fig. 1.

### *Electrochemical measurements with AC*_*60*_*/GCEs*

Electrochemical measurements were carried out by means of differential pulse voltammetry (DPV) by means of a PalmSens4 potentiostat (PalmSens BV, Houten, The Netherlands), using a previous accumulation step of the analyte on the electrode. First, the AC_60_/SPCE was introduced into the top of a sealed cell containing 1 mL of supporting electrolyte solution (pH 2.3), except for the optimization process, and the corresponding analyte concentration. The solution was stirred during 360 s at 70 °C. After this incubation time, in which the 4-ethyguaicol present in the gas phase over this solution was accumulated on the working electrode, differential pulse voltammograms were performed in a drop of 100 µL of supporting electrolyte solution deposited on the AC_60_/GCE.

## Results

The electrochemical oxidation of 4-ethylguaicol to the corresponding quinone has been described as a process which involves the previous formation of an unstable phenoxy radical in a one-electron and one-proton step [23, 30]. This oxidation response gives rise to an analytical signal suitable for the sensitive determination of this compound using conventional electrodes. However, it may show a lack of selectivity when the analysis of 4-ethylguaicol is carried out on complex samples. The modification of the working electrode with a molecularly imprinted polypyrrole polymer [26] and with C_60_ [27] has therefore been studied to avoid this possible loss of selectivity. Better results were achieved in the case of the nanomaterial, so they are the only ones that will be shown below. In this case, SPCEs were selected considering their better properties to those of conventional electrodes, including their low cost that allows the simple production of a large number of disposable devices with numerous possibilities of modification and, in addition, their ease of adaptation to small portable instrument systems [31].

The oxidation of 4-ethylguaicol to its corresponding quinone may be observed using an AC_60_/SPCE by means of DPV in a 100 µL droplet dropped of supporting electrolyte directly onto the three-electrode system, after a previous accumulation of the analyte on the electrode surface in the gas phase, to increase the selectivity of the analytical method. This oxidation signal was influenced by different parameters, including temperature and incubation time applied during the accumulation step, as well as pH of the supporting electrolyte solution used for accumulation and DPV measurements. Thus, in order to ensure that the analytical measurements were carried out under the best possible conditions, an optimization stage of these variables was first performed. Experimental designs were used as a tool for optimization which allow to explore a wide experimental range with a reduced number of experiments. Moreover, they are more effective than the “one-at-time” experiments being able to detect interactions between the different factors that could lead to wrong decisions [32]. A 2^3^ central composite experimental design was then performed, taking the following values as high, low and central levels for each of the factors to be optimized, being the oxidation intensity obtained for a 0.98 mg/L 4-ethylguaiacol solution the response to be optimized (Table 2).

**Table 2:** Factors optimized in the 2^3^ central composite experimental design

	High level	Central level	Low level
pH	5	4	3
Incubation time (s)	600	360	120
Incubation temperature (°C)	60	45	30

**Fig. 2:**
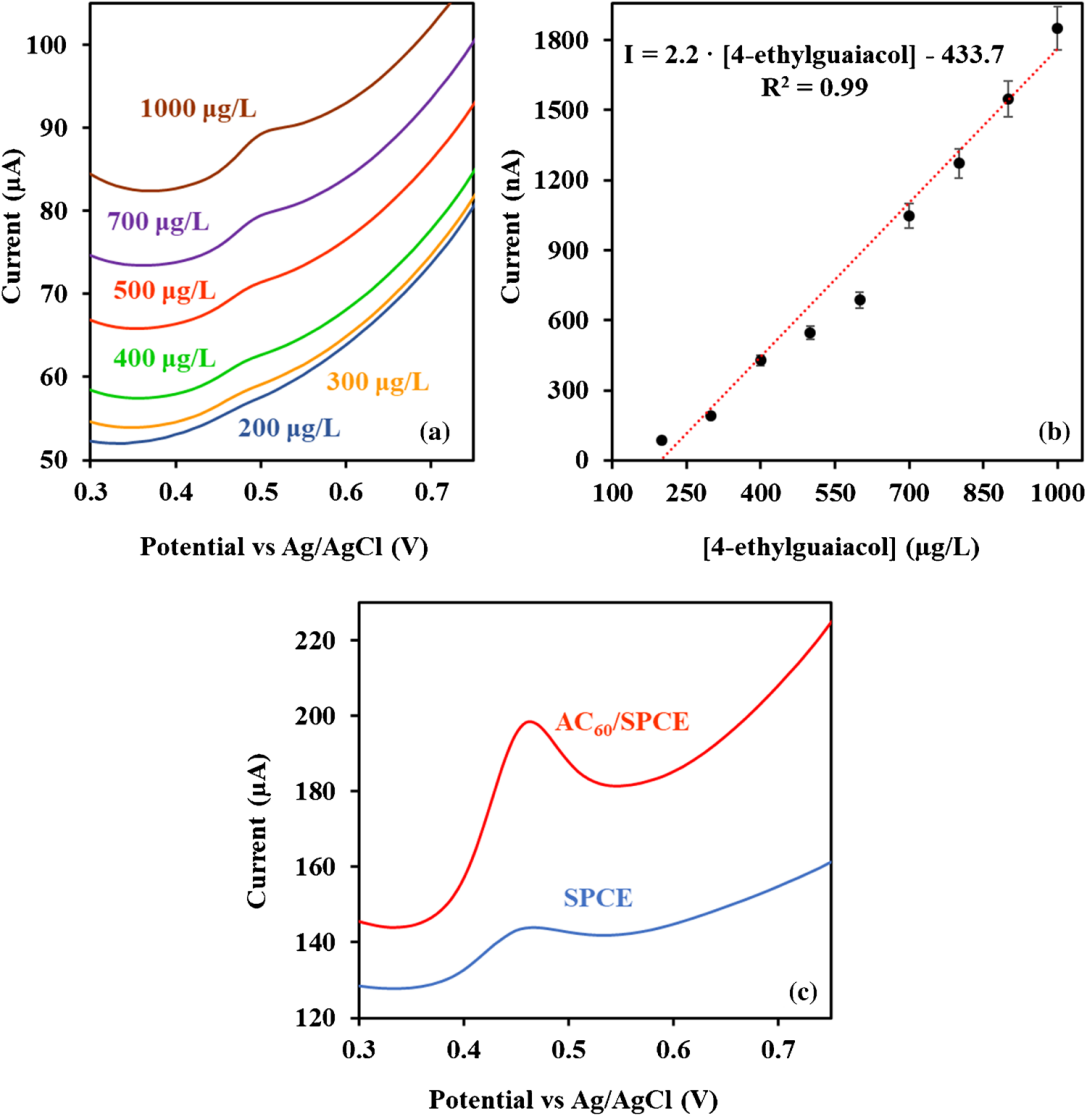
**a** DPV curves and **b** experimental points and calibration plot obtained under optimized experimental conditions in the 4-ethylguaicol concentration ranging from 200 to 1000 µg/L using an AC_60_/SPCE. **c** DPV curves obtained for a 2 mg/L 4-ethylguaicol solution using different electrodes (phosphate; pH, 2.3; incubation time, 12 min; incubation temperature, 70 °C; pulse potential, + 0.2 V; step potential, + 0.01 V; pulse time, 0.02 s; and scan rate, 50 mV/s)

Seventeen experiments were consequently performed with different combinations of these values, including three replicates in the central point to evaluate the residual error (Table 3). From the analysis of the results obtained for the different experiments, the following optimum values for the variables were found: pH, 2.3; incubation time, 12 min; and incubation temperature, 70 °C, using Statgraphics program [33]. Under these optimized conditions, an oxidation peak was observed at a potential of + 0.48 V vs. Ag/AgCl, which increased with the increasing analyte concentration (Fig. 2a, b) and being the sensitivity of the AC_60_/SPCEs much higher than that obtained using bare SPCEs (Fig. 2c). Thus, the modification of the electrode surface by an activated film of C_60_ notably improves the reactivity of a SPCE.

**Table 3:** The 2^3^ experimental design for optimization of experimental variables in 4-ethylguaicol determination using AC_60_/SPCEs

Incubation temperature (°C)	pH	Incubation time (s)
30	3	120
60	3	120
30	5	120
60	5	120
30	3	600
60	3	600
30	5	600
60	5	600
19	4	360
70	4	360
45	2.3	360
45	5.7	360
45	4	35
45	4	684
45	4	360
45	4	360
45	4	360

**Fig. 3:**
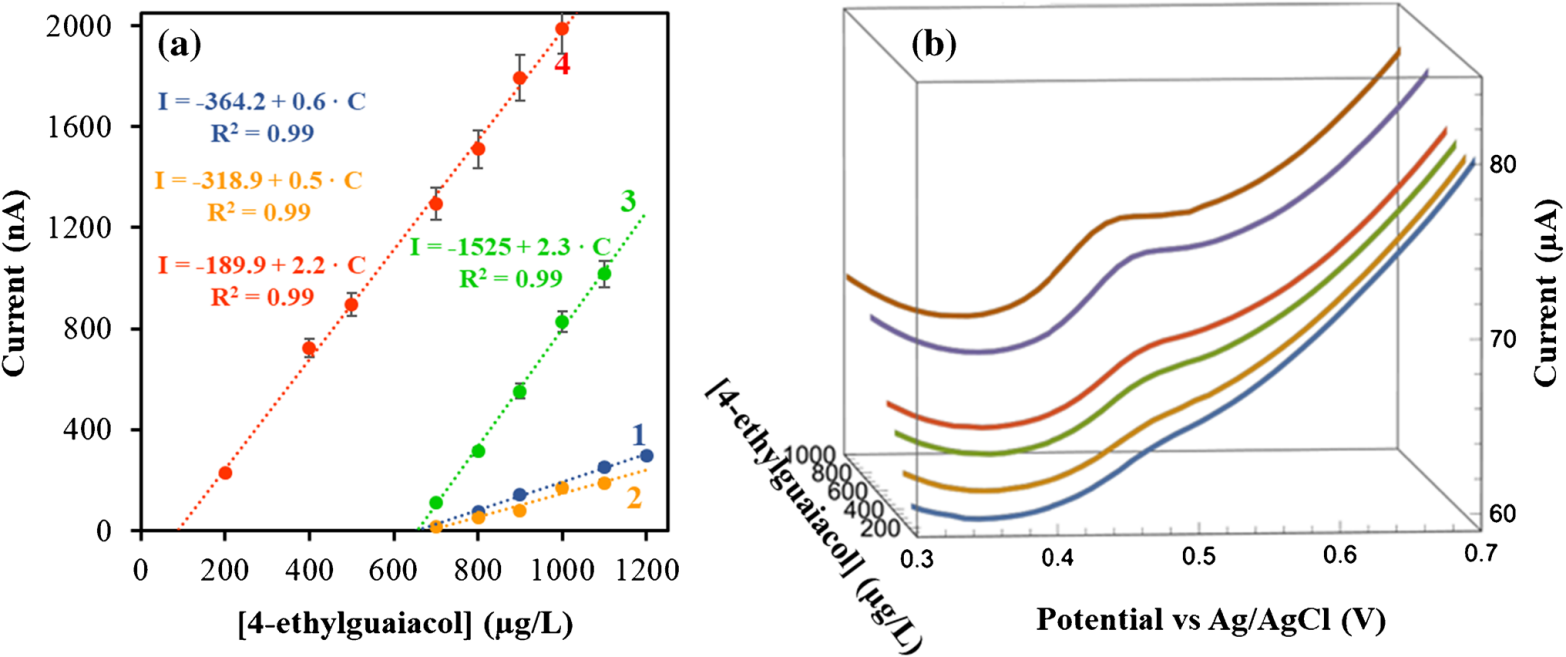
**a** Experimental points and calibration plots obtained using an AC_60_/SPCE for different incubation times ((1) 3 min [blue], (2) 4 min [yellow], (3) 5 min [green] and (4) 6 min [red]). **b** DPV curves obtained in the 4-ethylguaicol concentration ranging from 200 to 1000 µg/L using an AC_60_/SPCE (phosphate; pH, 2.3; incubation time, 6 min; incubation temperature, 70 °C; pulse potential, + 0.2 V; step potential, + 0.01 V; pulse time, 0.02 s; and scan rate, 50 mV/s)

An incubation time of 12 min implies spending excessively time in the construction of each calibration set, which is difficult to adapt for real-time analysis. Thus, a deep study of the influence of incubation time on the sensitivity value of the method was carried out. Different calibration sets were constructed in the concentration range between 200 and 1000 µg/L, using different incubation times ranging from 3 to 6 min. As it can be seen in Fig. 3a, the sensitivity value found for 6 min was similar to that obtained for 12 min. Thus, this value was selected for next experiments, obtaining as well defined DPV signals for the quantification of 4-ethylguaicol (Fig. 3b).

Finally, the developed procedure based on AC_60_/SPCEs was validated under the optimized conditions for the 4-ethylguaicol determination by means of the estimation of its precision, capability of detection, decision limit and trueness. Hence, different calibration plots were built using ordinary linear regressions in the concentration ranging from 200 to 1000 µg/L. Outlier points, characterized by a studentized residual higher than 2.5, in absolute value, were rejected with the aim to achieve a perfect evaluation of the different calibration parameters [33]. The precision of the method was then assessed by studying the reproducibility obtained for the slopes of three different validated calibration sets, in order to evaluate the dispersion referred to a range of concentrations instead of just a single concentration value. The value of relative standard deviation (RSD) obtained was 7.6%, showing a high degree of precision.

Capability of detection (CC_*β*_) and decision limit (CC_*α*_) were calculated using DETARCHI program [34], according to the ISO 11843 approach, based on a linear regression model [35]. A value of 58.7 µg/L was obtained for CC_*α*_, for a probability of false positive (*α*) and negative (*β*) of 0.05. The value found for CC_*β*_ was inferior than that of the concentration used in the first calibration point, so 200 µg/L was taken as the capability of detection of the method.

### Interference analysis

**Fig. 4:**
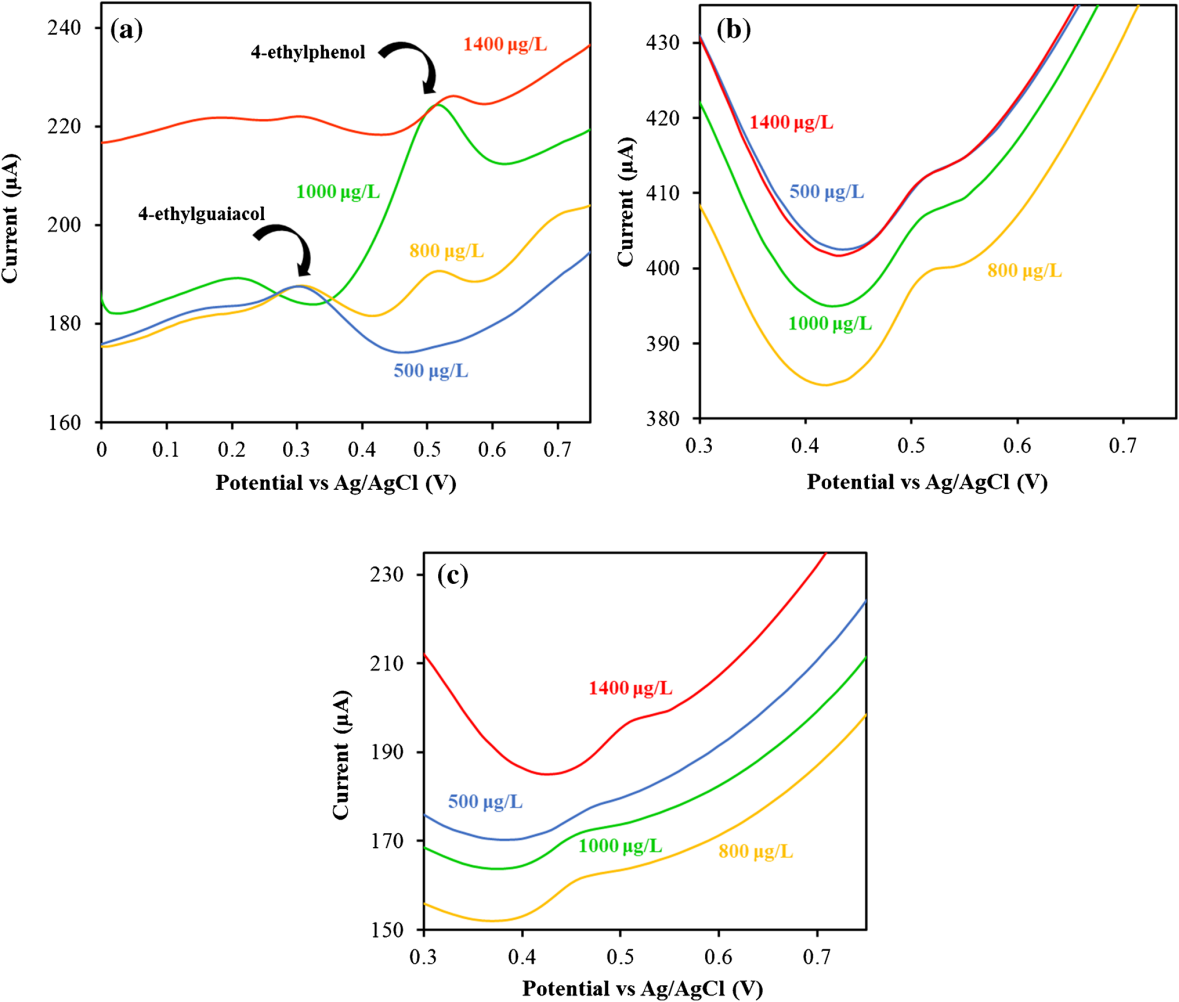
DPV curves obtained for solutions containing 700 µg/L of 4-ethylguaicol and different concentrations of **a** 4-ethylphenol, **b** 4-vinylphenol and **c** *p*-coumaric acid using an AC_60_/SPCE (phosphate; pH, 2.3; incubation time, 6 min; incubation temperature, 70 °C; pulse potential, + 0.2 V; step potential, + 0.01 V; pulse time, 0.02 s; and scan rate, 50 mV/s)

The selectivity towards 4-ethylguaicol of the developed AC_60_/SPCEs was also analysed by studying the possible interference caused by the presence of other compounds with a similar structure, including 4-ethylphenol, 4-vinylphenol and *p*-coumaric acid. Thus, different solutions of the possible interferent with concentrations between 500 and 1400 µg/L were analysed, keeping the concentration of 4-ethylguaicol at a constant value of 700 µg/L in all of them. Some grade of interference in the determination of 4-ethylguaicol was found for 4-ethylphenol at concentrations higher than 800 µg/L, due to the high overlap of the oxidation peaks of both phenols (Fig. 4a). 4-Vinylphenol and *p*-coumaric acid showed no influence on the oxidation signal of 4-ethylguaicol even at high concentration levels (1400 µg/L) as it can be seen in Fig. 4b and c.

### Wine sample analysis

The developed AC_60_/SPCE sensors were also validated in terms of trueness by means of their application to the analysis of 4-ethylguaicol in different wine samples. Two commercial samples of red wine (Tempranillo variety) and two of white wine (Airen and Verdejo varieties) were studied, not finding the presence of 4-ethylguaicol in any of them. Thus, an analysis of spiked wine samples was performed by means of standard addition method, obtaining results far from the concentration with which the samples were enriched. Hence, an important matrix effect was observed, which was avoided using a liquid–liquid extraction procedure for each standard: 2 mL of diethyl ether was added to 5 mL of spiked wine sample and ultrasonicated for 5 min. Then, 100 µL of the ether layer was transfer to a vial containing 900 µL of pH 2.3 phosphate buffer solution for the accumulation step. The DPV analysis of the extracted phases according to this standard addition procedure gave rise to results shown in Table 4. As it can be seen, there is significant agreement of the results obtained by the described procedure and the real content of 4-ethylguaicol of the spiked wine samples, obtaining good recovery percentages between 96 and 106% which confirm the potential of the developed sensors for practical applications.

**Table 4:** Determination of 4-ethylguaiacol in different wine samples by DPV using an AC_60_/SPCE

Sample	Concentration added (µg/L)	Concentration found (µg/L)	Recovery (%)
White wine 1	–	< CC_*β*_	–
	250	265 ± 22	106
	375	380 ± 7.2	101
	500	495 ± 22	99
White wine 2	–	< CC_*β*_	–
	250	247 ± 20	99
	375	368 ± 6.4	98
	500	524 ± 37	105
Red wine 1	–	< CC_*β*_	–
	250	240 ± 19	96
	375	369 ± 12	98
	500	518 ± 69	104
Red wine 2	–	< CC_*β*_	
	250	257 ± 22	103
	375	386 ± 38	103
	500	504 ± 56	101

## Conclusions

This work describes an electrochemical method that combines the important advantages of SPCEs, related to its low cost and possibility of adaptation to in situ analysis, with the use of nanomaterials such as C_60_ for its modification, which provides improved sensitivity in the analysis of 4-ethylguaiacol. In addition, the prior accumulation of the analyte present in the gas phase on the electrode surface manages to improve selectivity, allowing its analysis in the range of concentrations normally present in wine. The developed sensors are then presented as an interesting alternative to the more complex and expensive classical techniques used by wine producers for the analysis of 4-ethylguaiacol.
